# Deubiquitinase YOD1 Inhibition Suppresses DEX‐ and Denervation‐Induced Muscle Atrophy Through MAFbx Destabilization

**DOI:** 10.1002/jcsm.70300

**Published:** 2026-04-29

**Authors:** Jongbeom Chae, Seon Min Woo, Seung Un Seo, Simmyung Yook, Hyo Je Cho, Jung Yeol Lee, Kyoung jin Min, Junhyeon Park, Dongryeol Ryu, Taeg Kyu Kwon

**Affiliations:** ^1^ Department of Immunology, School of Medicine Keimyung University Daegu South Korea; ^2^ School of Pharmacy Sungkyunkwan University Suwon Gyeonggi South Korea; ^3^ Department of Biochemistry Chungbuk National University Cheongju South Korea; ^4^ New Drug Discovery Center (NDDC), Daegu Gyeongbuk Medical Innovation Foundation (K‐MEDI Hub) Daegu South Korea; ^5^ Department of Biomedical Science and Engineering Gwangju Institute of Science and Technology (GIST) Gwangju South Korea; ^6^ Center for Forensic Pharmaceutical Science Keimyung University Daegu South Korea

**Keywords:** deubiquitinase, MAFbx, muscle atrophy, ubiquitin proteasome system, YOD1

## Abstract

**Backgrounds:**

Muscle atrophy, characterized by progressive loss of muscle mass and function, is driven by muscle‐specific E3 ligases MAFbx and MuRF1. While transcriptional regulation of E3 ligases is documented, the mechanism of their regulation by the ubiquitin–proteasome system remains unclear. This study aims to identify a deubiquitinase (DUB) regulating these E3 ligases and reveal the mechanisms underlying the mitigation of muscle atrophy through inhibition of the discovered DUB.

**Methods:**

Differentiated C2C12 myotubes were screened using siRNAs to identify DUB genes that can regulate muscle atrophy. Muscle fibre cross‐sectional area (CSA), grip strength and gene expression (MAFbx, MyoD, etc.) were evaluated in muscle atrophy‐induced mouse model. Human translational relevance was analysed using GTEx skeletal muscle data.

**Results:**

We identified that OTU DUBs family genes are increased (log2 FC > 1, *p* < 0.05) in DEX‐induced muscle atrophy. Pharmacological (ubiquitin isopeptidase inhibitor I, G5) and genetic inhibition of YOD1 alleviated DEX‐ and denervation‐induced muscle atrophy by MAFbx destabilization. The UBX domain of YOD1 was found to interact with the LZ domain of MAFbx, and YOD1 stabilized the MAFbx protein by removing polyubiquitin chains at lysine 48 in MAFbx. In in vivo mouse models, G5 treatment effectively ameliorated DEX‐ or NTX‐induced muscle atrophy. Specifically, G5 increased grip strength by 37.64% (DEX, *p* < 0.0001) and 36.37% (NTX, *p* < 0.01), while muscle fibre size was improved by 35.85% (DEX, *p* < 0.01) and 30.76% (NTX, *p* < 0.0001). These improvements were accompanied by the restoration of MyoD and eIF3‐f expression. Consistently, GTEx‐based analysis revealed that high YOD1 expression in human skeletal muscles is significantly associated with an increased proportion of smaller fibres (< 2000 μm^2^), correlating with enriched proteostasis (NES = 1.51)‐related and muscle development (NES = −1.44)‐related transcriptional signatures.

**Conclusions:**

Our study indicates that YOD1 inhibition destabilizes MAFbx protein levels, leading to protection against DEX‐ and denervation‐induced muscle atrophy. Integration of human GTEx data further supports the translational relevance of YOD1 as a regulator of muscle fibre homeostasis. This study provides new insights into the post‐translational regulation of muscle‐specific E3 ligases and presents evidence showing that targeting YOD1 is a promising therapeutic approach for the prevention and treatment of muscle atrophy.

## 1 Introduction

The maintenance of skeletal muscle is dependent upon the precise regulation of protein synthesis and degradation. However, when this balance is disrupted in various metabolic conditions, such as denervation, obesity, aeging and cancer, skeletal muscle atrophy ensues [1] [S1]. Atrophy is characterized by reduced proteins, degradation of myofibrils and a consequent loss of muscle mass and strength. The ubiquitin‐proteasome system (UPS) is the central mechanism mediating the degradation of key structural and regulatory proteins in myocytes [2]. The UPS regulates protein homeostasis through a process involving three key enzymes: ubiquitin‐activating E1 enzymes, ubiquitin‐conjugating E2 enzymes and ubiquitin E3 ligases [3]. During muscle atrophy, upregulation of muscle‐specific E3 ligases (MAFbx and MuRF1) promotes myofibrillar protein degradation, contributing to muscle wasting [4]. In atrophy models, these E3 ligases are transcriptionally regulated by diverse factors [5, 6]. Forkhead box O (FoxO) is a crucial transcription factor that upregulates MAFbx and MuRF1 expression by binding to their promoters [7]. In addition, because the promoter region of these E3 ligases contains the KLF15 binding site, glucocorticoids either directly regulate KLF15 or indirectly enhance KLF15‐mediated FoxO activation, thereby promoting the transcriptional regulation of MAFbx and MuRF1 [8]. Furthermore, C/EBPβ plays a critical role in skeletal muscle biology, particularly through its regulation of satellite cell activity and myogenic differentiation [9]. Although transiently expressed during the early phase of muscle regeneration, sustained or aberrant C/EBPβ expression suppresses myogenic differentiation and promotes muscle atrophy via activation of catabolic pathways under inflammatory conditions [10]. C/EBPβ increases *MAFbx* and *MuRF1* mRNA expression at the transcriptional level, thereby inducing muscle atrophy [11]. Moreover, previous studies reported that multiple transcription factors, such as NF‐κB, Smad2/3 and TFEB, can transcriptionally activate MAFbx and MuRF1 expression under atrophy conditions [12, 13, 14]. Although the transcriptional regulation of muscle‐specific E3 ligases is well‐documented, the mechanisms underlying their post‐translational regulation remain unclear.

Deubiquitinases (DUBs) are enzymes that cleave ubiquitin chains from substrate proteins and stabilize target proteins by preventing proteasomal degradation. They are divided into seven groups based on their catalytic domains: ubiquitin‐specific protease (USP), ovarian tumour proteases (OTU), ubiquitin C‐terminal hydrolase (UCH), Machado–Josephin disease (MJD), JAB1/MPN/Mov34 metalloprotease (JAMM), Ub‐containing novel DUB family (MINDY) and zinc finger–containing ubiquitin peptidase (ZUP) classes [15]. In skeletal muscles, DUBs contribute to various aspects of muscle physiology and development, including inflammation, proliferation, differentiation and regeneration [16]. The USP subfamily of DUBs plays a key role in myogenesis, differentiation and muscle wasting. USP1 knockdown attenuates muscle wasting by inactivating Akt/FoxO signalling under acute starvation conditions [17]. Moreover, muscle‐specific USP21 knockout (KO) mice increase muscle mass through improved mitochondrial function and fatty acid oxidation [18]. USP19 is upregulated in numerous mouse models of cancer, cachexia, denervation, fasting and diabetes [19, 20]. USP19 depletion increases myofibrillar protein expression, leading to the alleviation of dexamethasone (DEX)‐ and denervation‐induced muscle atrophy [21]. However, USP19 KO downregulates *MAFbx* and *MuRF1* mRNA expression [22] and its specific target proteins remain unknown. Despite the significance of DUBs in muscle health, the specific substrates of each DUB and the detailed mechanisms are not fully understood.

Therefore, the aim of our study was to identify novel DUBs that regulate skeletal muscle and investigate their specific substrates in muscle atrophy.

## 2 Methods

### 2.1 Cell Culture

C2C12 myoblast cell line (CRL‐1772, ATCC, VA, USA) was cultured in growth medium (GM; DMEM‐H supplemented with 10% FBS) in a humidified atmosphere containing 5% CO_2_ at 37°C.

### 2.2 Induction of Myogenic Differentiation and DEX‐Induced Muscle Atrophy In Vitro

C2C12 cells were seeded in a six‐well plate and cultured to 80%–90% confluence followed by culture in differentiation induction medium (DM) containing 2% horse serum for 2 or 6 days. To evaluate the effect on muscle atrophy, 5‐day differentiated C2C12 myotubes were treated with 50 μM of DEX and indicated concentrations of G5 (a ubiquitin isopeptidase inhibitor) for 48 h.

### 2.3 Cell Transfections

DUB siRNAs and plasmids were obtained from Bioneer (Daejeon, Korea). C2C12 cells were transfected with 30 nM control or target siRNAs using Lipofectamine RNAiMAX (Thermo Fisher Scientific) in Opti‐MEM (Gibco). For plasmid transfection, C2C12 cells were transfected with 2 μg of plasmid DNA using Lipofectamine 2000 reagent (Thermo Fisher Scientific). The medium was replaced with complete growth medium 7 h after transfection. The siRNAs and plasmids are listed in Table S1.

### 2.4 Immunofluorescence Staining for C2C12 Myotubes

C2C12 myoblasts (1 × 10^6^) were seeded onto eight‐chamber slides (SPL 30108, SPL LIFE SCIENCE, Korea) and incubated until 80%–90% confluence. The medium was replaced with DM. Cells were fixed with 4% PFA, permeabilized by 0.25% Triton X‐100, blocked using 1% BSA in PBS and incubated with Alexa Fluor 546‐conjugated anti‐MyHC (SCBT, CA, USA). Images were captured and processed using a fluorescence microscope (Leica, Wetzlar, Germany) and the ImageJ software (http://imagej.nih.gov/ij/).

### 2.5 Western Blotting

Western blotting was performed as previously described [S2]. Briefly, cells and tissues were washed with cold PBS and lysed on ice using a lysis buffer. After centrifugation at 10000 × *g* for 15 min at 4°C, supernatants were boiled with 5 × sample buffer, separated using SDS‐PAGE and transferred to nitrocellulose membranes. Proteins were probed with specific antibodies and visualized using ECL. Antibodies are listed in Table S2.

### 2.6 Giemsa Staining

Myoblasts were rinsed twice with cold PBS and fixed with 4% paraformaldehyde for 5 min. The cells were washed again and incubated with 10% Giemsa solution (Gibco) for 40 min. After two additional washes, the stained cells were visualized using a light microscope.

### 2.7 Reverse Transcription‐Quantitative Polymerase Chain Reaction (RT‐qPCR)

Total RNA was isolated using TRIzol reagent (Life Technologies; Gaithersburg, MD, USA), and cDNA was obtained using M‐MLV reverse transcriptase (Gibco‐BRL; Gaithersburg, MD, USA). SYBR Fast qPCR Mix (Takara Bio Inc., Shiga, Japan) was used for PCR, which was performed on a Thermal Cycler Dice Real Time System III (Takara Bio Inc.). Actin was used as the reference gene to calculate threshold cycle number (Ct), and ΔΔCt values of the genes were reported. The following primers were used to amplify the target genes. Plasmids are listed in Table S3.

### 2.8 Ubiquitination Assay

A ubiquitin assay was performed as previously reported [S2]. Briefly, cells were collected and rinsed with PBS containing 10 mM of N‐ethylmaleimide (NEM) to inhibit deubiquitination. They were then suspended in 90 μL of PBS/NEM with 1% SDS and heated at 95°C for 10 min. The resulting lysates were combined with lysis buffer containing 1 mM of PMSF and 5 mM of NEM, sheared using a 1‐mL syringe and centrifuged at 13000 × *g* for 10 min at 4°C. Supernatants were incubated overnight at 4°C with primary antibodies. After incubation, protein G agarose beads were added and rotated for 2 h to pull down immune complexes. The beads were washed twice with lysis buffer, resuspended in 2 × sample buffer, and boiled for 10 min. The eluted proteins were resolved using SDS‐PAGE, transferred onto nitrocellulose membranes, and the ubiquitinated proteins were visualized using a horseradish peroxidase (HRP)‐conjugated anti‐ubiquitin antibody.

### 2.9 Immunoprecipitation

Cells were lysed in CHAPS lysis buffer and incubated overnight with primary antibodies. Then, the lysates were treated with protein G agarose beads (Santa Cruz Biotechnology, Santa Cruz, CA, USA) for 2 h. After centrifugation, the supernatants were removed and the pellets were boiled in 2 × sample buffer. Protein interactions were detected using western blotting.

### 2.10 Animal Experiment

All mice were housed at 24°C ± 1°C with 10%–20% humidity, under a 12‐h light/dark cycle. Male C57BL/6 mice (5 weeks old) were obtained from JABio (Suwon, Korea). After a 1‐week acclimation period, experiments were initiated. Body weight was recorded every 2 days for the entire duration. On the final experimental day, the tibialis anterior (TA), extensor digitorum longus (EDL), soleus (SOL) and gastrocnemius (GAS) muscles were rapidly excised, weighed, consecutively numbered, frozen in liquid nitrogen and finally stored at −80°C. All animal experiments were approved by the Institutional Animal Care Committee of Keimyung University (approval number: KM‐2024‐11R1). The study was performed in compliance with ARRIVE guidelines, and all methods were implemented according to relevant guidelines and regulations.

### 2.11 DEX‐Induced Muscle Atrophy Model

Male C57BL/6 mice (6–8 weeks old) were continuously treated with DEX (20 mg/kg/day) via intraperitoneal (i.p.) injection for 3 weeks. In experiment, Ubiquitin isopeptidase inhibitor G5 (2 μg/kg/day) was injected intraperitoneally for 3 weeks. The control group was treated with sterile PBS only. No medication was administered to the control or DEX groups during the same period.

### 2.12 Sciatic Denervation‐Induced Muscle Atrophy Model

Denervation was performed by surgically removing the sciatic nerve from the right hind limb of male mice at the age of 8 weeks. In the experiment, G5 (2 μg/kg/day) was injected intraperitoneally for 2 weeks. The control group was treated with sterile PBS only. No medication was administered to the control or DEX groups during the same period.

### 2.13 Grip Strength Test

Grip strength was measured five times using a grip strength test machine (Grip Strength Meter for Mice and Rats, Ugo Basile Srl, Italy), according to the manufacturer's instructions. Results were standardized according to body weight, and average values were calculated.

### 2.14 Measurement of Cross‐Sectional Area (CSA)

Muscle tissues were fixed in 10% formalin, embedded in paraffin, cut into 4‐μm sections and stained with haematoxylin and eosin (H&E). Thereafter, the stained sections were observed under a light microscope. Myofiber CSA was quantified using ImageJ (v1.54g; Java 1.8.0_345, 64‐bit) using the Cross‐Sectional‐Analyser plugin and Stardist plugin.

### 2.15 Immunohistochemistry (IHC)

Mouse gastrocnemius tissues were fixed in 4% paraformaldehyde overnight at 4°C, dehydrated and embedded in paraffin, and 4‐μm sections were obtained. After deparaffinization and rehydration, antigen retrieval was performed by heating the sections in citrate buffer (pH 6.0) for 20 min. Endogenous peroxidase activity was blocked by incubating the slides with 3% hydrogen peroxide for 10 min. Sections were then blocked with 5% normal serum for 1 h at room temperature and incubated overnight at 4°C with YOD1 primary antibodies diluted in blocking buffer (1:1000). After washing, the sections were incubated with HRP‐conjugated secondary antibodies and developed using a DAB substrate kit. Stained tissues were visualized and imaged using a light microscope.

### 2.16 CSA Assessment and Gene Set Enrichment Analysis Using GTEx Transcriptome and Histology Data

The GTEx v8 skeletal‐muscle transcriptome (TPM matrix; gene_tpm_muscle_skeletal.gct, released September 11, 2020) and corresponding whole‐slide H&E images were analysed as previously reported [23] [S3, 4]. H&E slides were reviewed using Aperio ImageScope (v12.4.3.5008), and regions of interest (ROIs) containing transversely sectioned myofibers were cropped. Myofiber CSA was quantified using ImageJ (v1.54g; Java 1.8.0_345, 64 bits) using the Cross‐Sectional‐Analyser plugin. Pixel‐based measurements were converted to μm^2^ after calibration with the image scale, and regions in which fibre boundaries could not be reliably delineated were excluded. Donors were stratified into top 30 and bottom 30 groups according to YOD1 expression levels. CSA distributions were visualized using scatter plots, box plots, and histograms. Median differences between groups were tested using two‐sided Wilcoxon rank‐sum tests, and overall distributional differences across CSA bins were assessed using Pearson's *χ* ^2^ test of independence on pooled fibre counts.

For transcriptomic analysis, differential expression between the top 30 and bottom 30 YOD1 groups was evaluated using the Limma package (v3.58.1). The moderated log fold change was used as the pre‐ranked statistic for gene set enrichment analysis (GSEA). Multiple testing was performed using the Benjamini–Hochberg procedure to adjust for the false discovery rate (FDR). GSEA was performed using ClusterProfiler (v4.10.1) using the MSigDB C5 Gene Ontology collection retrieved via msigdbr (v24.1.0). Normalized enrichment scores (NES) were calculated, enrichment plots were generated using enrichplot (v1.22.0) and bubble plots were visualized using ggplot2 (v3.5.1). All analyses were performed using R (v4.3.2; RStudio v2023.12.1.402).

### 2.17 Statistical Analysis

The results are expressed as the mean ± standard deviation (SD) for in vitro studies and the mean ± standard error of the mean (SEM) for in vivo studies. Significant differences between groups were determined using one‐way ANOVA. Dunnett's post hoc test was used if the ANOVA indicated significance. All statistical analyses were performed using GraphPad Prism, version 9.0 (GraphPad Software, San Diego, CA, USA).

## 3 Results

### 3.1 YOD1 Is Upregulated in DEX‐Induced Muscle Atrophy In Vivo

**FIGURE 1:**
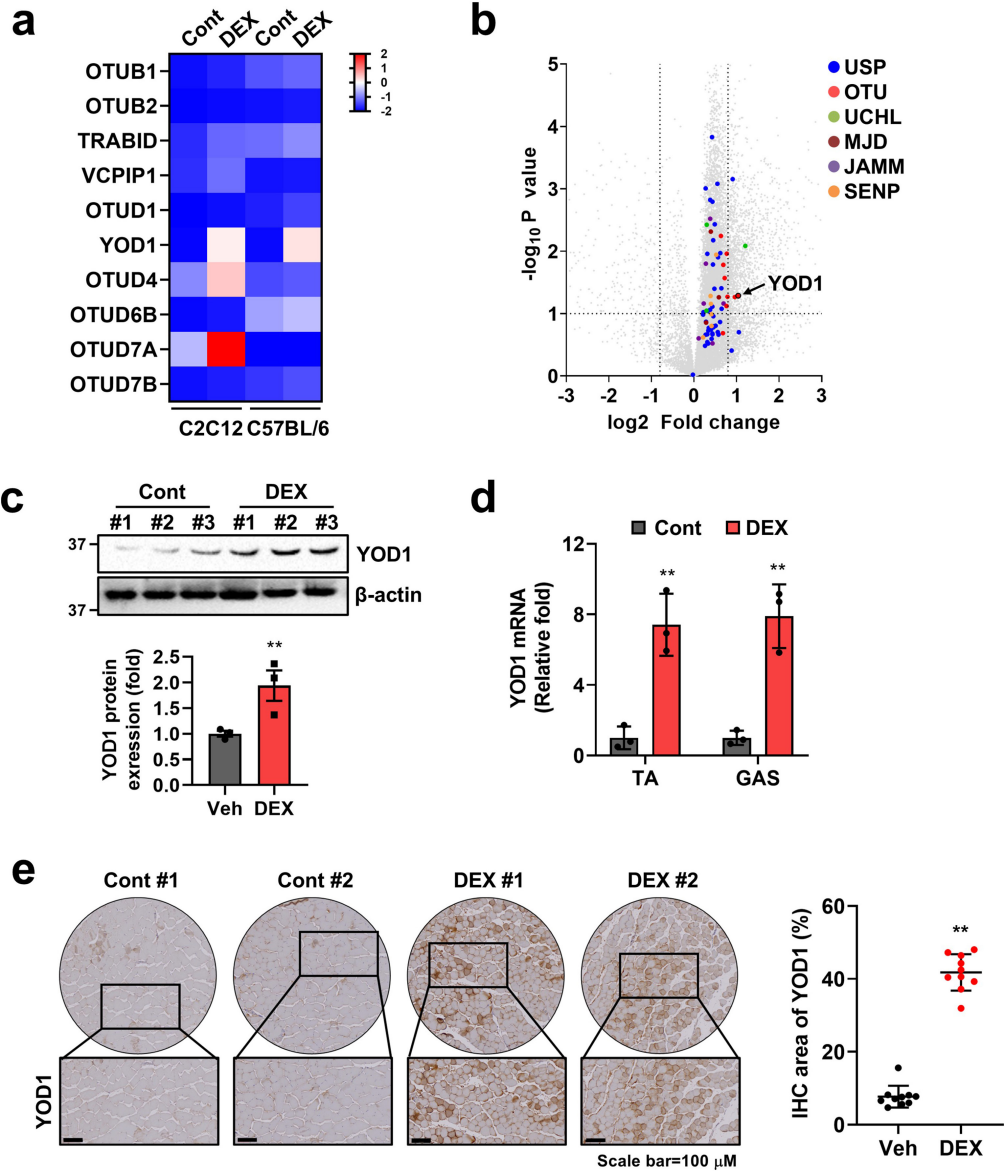
Screening of DUBs in DEX‐induced muscle atrophy and changes in YOD1 expression during muscle atrophy. (a) Heatmap generated from the analysis of GEO datasets, GSE159952 and GSE149453, used to identify OTU family DUBs. (b) Volcano plot of RNA‐Seq data, where the x‐axis represents the fold change. The *y*‐axis represents the log_10_ *p*‐value for each *x*‐axis point. YOD1 is marked. (c, d) Examination of protein and mRNA levels of YOD1 in GAS or TA muscle tissue of mice treated with or without dexamethasone (DEX) for 3 weeks. (e) Expression of YOD1 in the GAS muscle tissue of mice treated with or without DEX for 3 weeks measured using immunohistochemistry. Values in the graphs represent the mean ± standard deviation (SD) of three independent experiments. **p < 0.01 compared to vehicle.

We analysed the expression profiles of 73 DUBs using gene expression omnibus (GEO) datasets (GSE159952 and GSE149453) in DEX treatment, a synthetic glucocorticoid analogue [24, 25]. Among them, the expression of OTU family genes was consistently increased in both DEX‐treated muscle tissues and cells (Figures 1a,b and S1a). We further investigated the protein expression levels of the OTU DUBs family in the GAS muscle tissue of mice injected with DEX for 3 weeks. The results demonstrated the upregulation of YOD1, TRABID and OTUD7A expression and the downregulation of OTUD1, OTUD4 and OTUD6B expression (Figures 1c and S1b). Moreover, the YOD1‐positive area in the DEX‐induced muscle atrophy group was significantly higher than in the control group (Figure 1e). YOD1 protein expression was consistently upregulated in TA, EDL and SOL muscle tissues in DEX‐injected mice (Figure S1c). Moreover, the *YOD1* mRNA level was increased in DEX‐treated TA and GAS muscle tissues (Figure 1d). Furthermore, YOD1 was increased under various muscle atrophy‐inducing conditions in vitro, including palmitic acid (PA), TNF‐α, H_2_O_2_ and serum starvation (Figure S1d). These results indicate the importance of YOD1 in muscle atrophy.

### 3.2 YOD1 Inhibition Alleviates DEX‐Induced Myotube Atrophy

**FIGURE 2:**
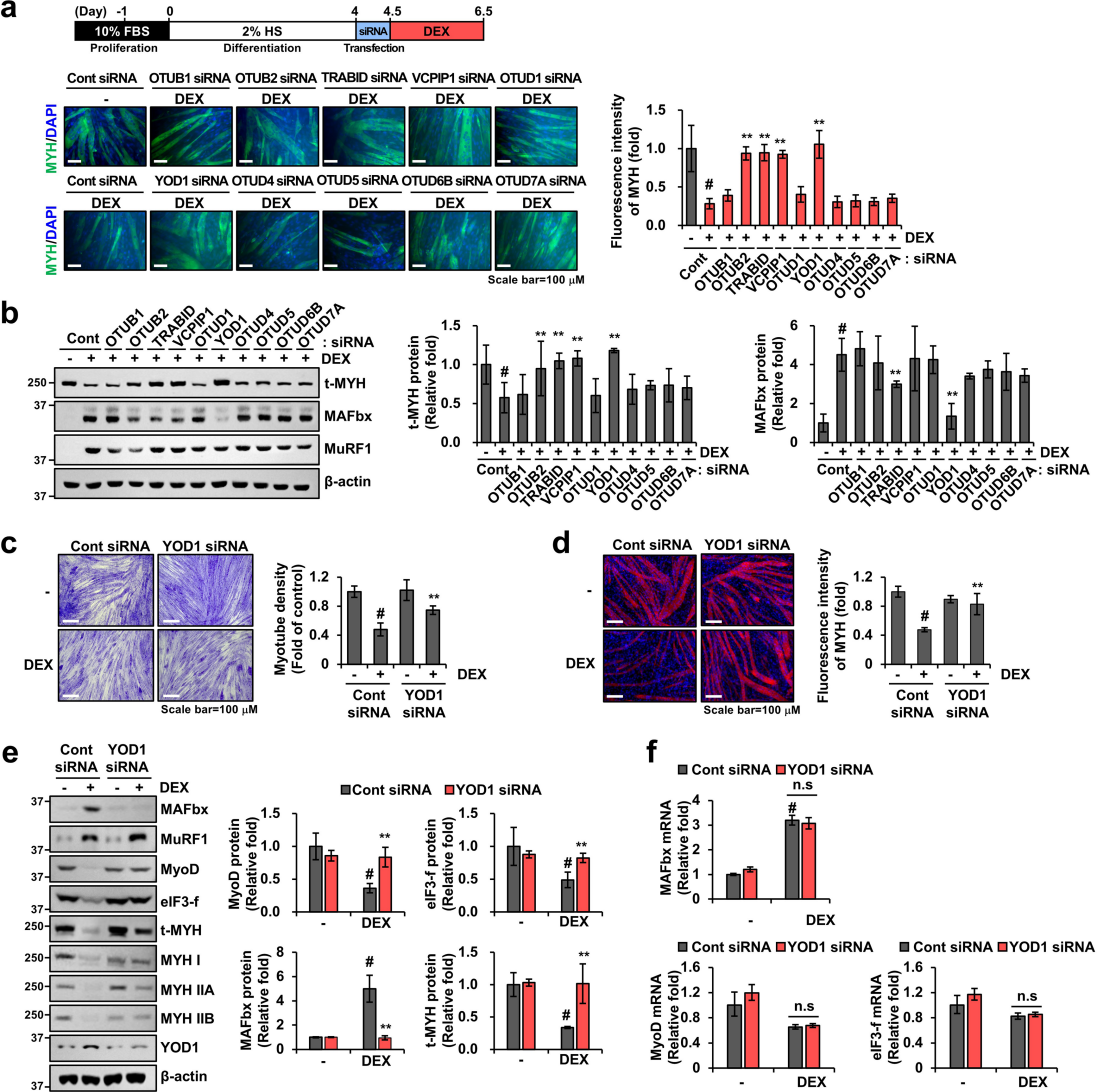
Effects of YOD1 silencing on DEX‐induced muscle atrophy in differentiated C2C12 myotubes. (a, b) C2C12 myotubes transfected with siRNA of each OTU family gene were treated with DEX for 48 h. Immunofluorescence (IF) was performed using an Alexa Fluor 488‐conjugated MYH antibody, and nuclei were stained with DAPI (a). Protein levels were determined using western blotting (b). (c–f) C2C12 myotubes transfected with control or YOD1 siRNA were treated with DEX for 48 h. Cells were fixed and stained with Giemsa (c). IF was performed using an Alexa Fluor 546‐conjugated MYH antibody, and nuclei were stained with DAPI (d). Protein (e) and mRNA (f) levels were determined using western blotting and qPCR, respectively. ^#^ *p* < 0.01 compared to the control. **p < 0.01 compared to DEX.

Differentiated C2C12 myotubes (4‐day postdifferentiation) were transfected with siRNAs targeting each OTU DUB gene, followed by DEX treatment (Figure 2a). OTUB2, TRABID, VCPIP1 and YOD1 knockdown alleviated the DEX‐induced reduction in myotube formation and myosin heavy chain (MYH) expression (Figure 2a,b). Notably, the DEX‐mediated upregulation of MAFbx, a muscle‐specific E3 ligase, was completely decreased by YOD1 knockdown, while another E3 ligase MuRF1 was not affected (Figure 2b). We found that YOD1 knockdown blocks the DEX‐induced decrease in myotube density (Figure 2c). DEX decreased the fluorescence intensity and protein expression of MYH and isoforms (t‐MYH, MYH I, MYH IIA and MYH IIB), whereas these alterations were attenuated by YOD1 deletion (Figure 2d,e). Although DEX increased the expression of both MAFbx and MuRF1, YOD1 knockdown only inhibited MAFbx protein levels, thereby reversing the degradation of MyoD and eIF3‐f expression, substrates of MAFbx (Figure 2e). YOD1 knockdown did not affect *MAFbx*, *MyoD* and *eIF3‐f* mRNA levels upon DEX treatment (Figure 2f). mTOR/Akt signalling has been implicated in protein synthesis, and DEX induces muscle atrophy by inhibiting protein synthesis [S5]. siRNA‐mediated YOD1 knockdown prevented DEX‐mediated dephosphorylation of Akt, p70S6K and 4EBP1, thereby maintaining their protein synthesis capacity (Figure S2a). These data suggest that knockdown of YOD1 alleviates DEX‐induced muscle atrophy by inhibiting MAFbx‐dependent MyoD and eIF3‐f degradation.

### 3.3 YOD1 Deubiquitinates and Stabilizes MAFbx

**FIGURE 3:**
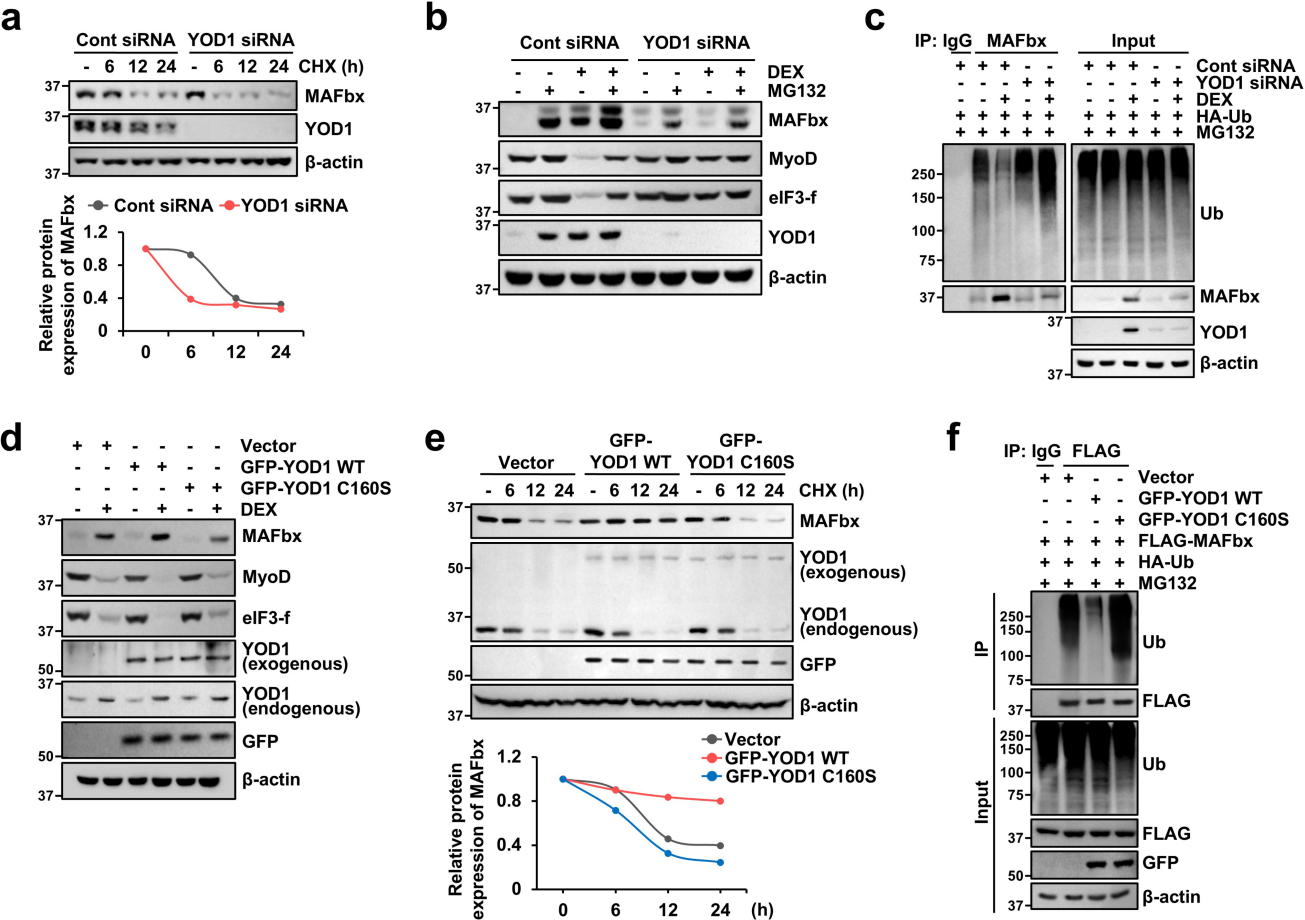
YOD1 deubiquitinases and stabilizes MAFbx. (a) C2C12 myotubes were transfected with control or YOD1 siRNA and then treated with 20 μg/mL of cycloheximide (CHX) for the indicated durations. (b) C2C12 myotubes were transfected with control or YOD1 siRNA and treated with 0.25 μM of MG132, followed by DEX treatment for 12 h. (c) To analyse the ubiquitination of endogenous MAFbx, C2C12 myotubes were co‐transfected with control or YOD1 siRNA in the presence of HA‐Ub and treated with 0.25 μM of MG132, followed by DEX treatment for 24 h. Ubiquitination of endogenous MAFbx was detected using the ubiquitination assay. (d,e) C2C12 myotubes were transfected with vector, GFP‐YOD1 WT or GFP‐YOD1 C160S plasmid and then treated with DEX (d) or 20 μg/mL of CHX (e) for the indicated durations. (f) C2C12 myoblasts were co‐transfected with vector, GFP‐YOD1 WT or GFP‐YOD1 C160S plasmid in the presence of HA‐Ub and FLAG‐MAFbx and treated with MG132 for 12 h. Ubiquitination of exogenous MAFbx was detected using the ubiquitination assay. The band intensity of MAFbx was analysed using ImageJ.

YOD1 knockdown resulted in the rapid degradation of MAFbx protein in the presence of cycloheximide (CHX), a protein synthesis inhibitor (Figure 3a). Additionally, MG132 (a proteasome inhibitor) suppressed DEX‐induced MAFbx degradation following YOD1 knockdown (Figure 3b). Furthermore, DEX inhibited MAFbx ubiquitination, whereas YOD1 depletion enhanced MAFbx ubiquitination (Figure 3c). We investigated the effect of a catalytic inactive mutant (C160S) of YOD1. Overexpression of YOD1 wild‐type (WT) sustained DEX‐mediated MAFbx upregulation compared to that in DEX‐treated vector cells, whereas overexpression of YOD1 C160S showed the opposite effect (Figure 3d). Depending on the alteration of MAFbx, MyoD and eIF3‐f degradation under DEX treatment was greater in cells expressing YOD1 WT than in those expressing YOD1 C160S (Figure 3d). Moreover, YOD1 WT resulted in persistent MAFbx expression following CHX treatment, whereas YOD1 C160S resulted in a rapid decrease of MAFbx expression after 12 h (Figure 3e). Furthermore, ubiquitination of MAFbx was decreased by YOD1 WT and increased by YOD1 C160S (Figure 3f). These results indicate a critical role for YOD1 in MAFbx ubiquitination and stabilization.

### 3.4 YOD1 Interacts With MAFbx and Eliminates Polyubiquitin Chains at K48 Residue of MAFbx

**FIGURE 4:**
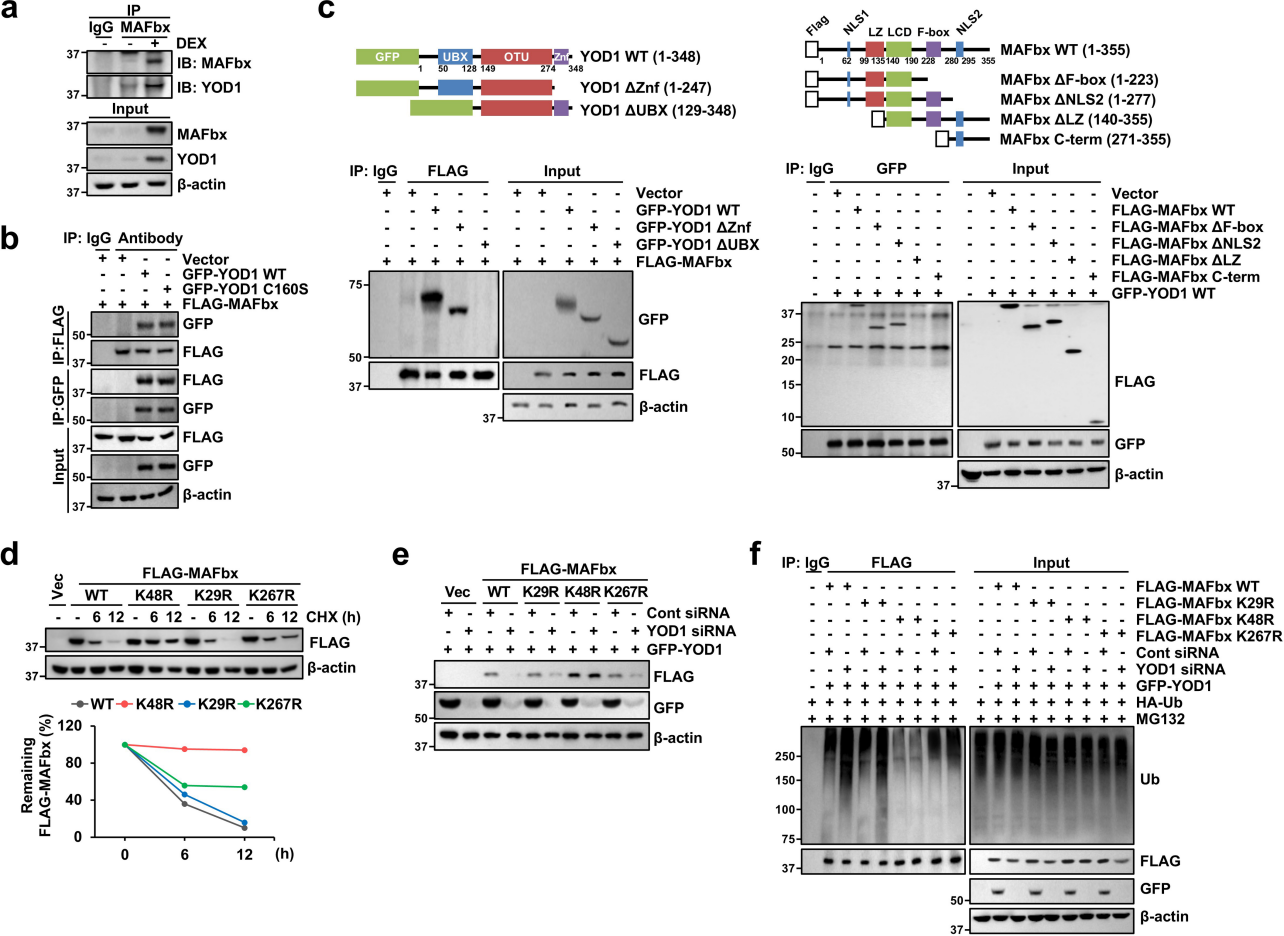
YOD1 interacts with MAFbx and removes polyubiquitin chains at K48 of MAFbx. (a) C2C12 myotubes were treated with or without DEX for 48 h. Cell lysates were immunoprecipitated with an anti‐MAFbx antibody, followed by immunoblotting (IB) with anti‐YOD1 or anti‐MAFbx antibodies. (b) C2C12 myoblasts were co‐transfected with vector, GFP‐YOD1 WT, or GFP‐YOD1 C160S in the presence of FLAG‐MAFbx. Interactions were demonstrated using IP. (c) C2C12 myoblasts were co‐transfected with vector, GFP‐YOD1 WT, GFP‐YOD1 ΔZn, or GFP‐YOD1 ΔUBX in the presence of FLAG‐MAFbx (left panel). C2C12 myoblasts were co‐transfected with vector, FLAG‐MAFbx WT, FLAG‐MAFbx ΔF‐box, FLAG‐MAFbx ΔNSL2, FLAG‐MAFbx ΔLZ, or FLAG‐MAFbx c‐terminal in the presence of GFP‐YOD1 WT (right panel). Interactions were demonstrated using IP. (d) C2C12 myoblasts were transfected with vector, FLAG‐MAFbx WT, FLAG‐MAFbx K29R, FLAG‐MAFbx K48R, or FLAG‐MAFbx K267R and then treated with 20 μg/mL of CHX for the indicated durations. The band intensity of FLAG was analysed using ImageJ. (e, f) C2C12 myoblasts were co‐transfected with vector, FLAG‐MAFbx WT, FLAG‐MAFbx K29R, FLAG‐MAFbx K48R or FLAG‐MAFbx K267R in the presence of control or YOD1 siRNA. The protein level (e) and ubiquitination of exogenous MAFbx (f) was measured using western blotting and ubiquitination assay, respectively.

Immunoprecipitation (IP) assay showed that endogenous YOD1 binds to endogenous MAFbx, and that this interaction was significantly increased by DEX (Figure 4a). Notably, the catalytically inactive mutant retained its ability to interact with MAFbx, indicating that their interaction is independent of YOD1 DUB activity (Figure 4b). To further define the critical regions for this interaction, we generated several YOD1 and MAFbx truncation mutants. YOD1 consists of an N‐terminal ubiquitin regulatory X (UBX) domain, a central OTU domain and a C‐terminal C2H2‐type zinc finger (Znf) domain. The results demonstrated that MAFbx binds to N‐terminal (ΔZnf) but not C‐terminal (ΔUBX) of YOD1, suggesting an interaction between the UBX domain of YOD1 and MAFbx (Figure 4c). MAFbx contains four major domains: the nuclear localization signal (NLS), leucine zipper (LZ) domain, leucine‐charged residue‐rich domain (LCD) and F‐box domain. The ΔLZ and C‐terminal domains of MAFbx failed to bind with YOD1, indicating that the LZ domain of MAFbx is associated with YOD1‐MAFbx interaction (Figure 4c).

Next, we surveyed three lysine residues (K29, K48 and K267) mutated to arginine to identify the specific lysine residues of MAFbx regulated by YOD1‐mediated polyubiquitination. K48 and K267 mutants sustained MAFbx expression by CHX, whereas the K29 mutant supported MAFbx degradation, similar to MAFbx WT, in CHX‐treated cells (Figure 4d). Interestingly, WT, K29R and K267R decreased the exogenous MAFbx expression following YOD1 knockdown, whereas K48R had no effect (Figure 4e). Additionally, YOD1 depletion increased MAFbx ubiquitination in WT, K29R and K267R MAFbx; however, K48R mutation significantly decreased MAFbx ubiquitination (Figure 4f). Taken together, these findings suggest that YOD1 directly interacts with MAFbx and inhibits the polyubiquitination of MAFbx at K48.

### 3.5 Effect of the YOD1 Inhibitor G5 on DEX‐Induced Myotube Atrophy

**FIGURE 5:**
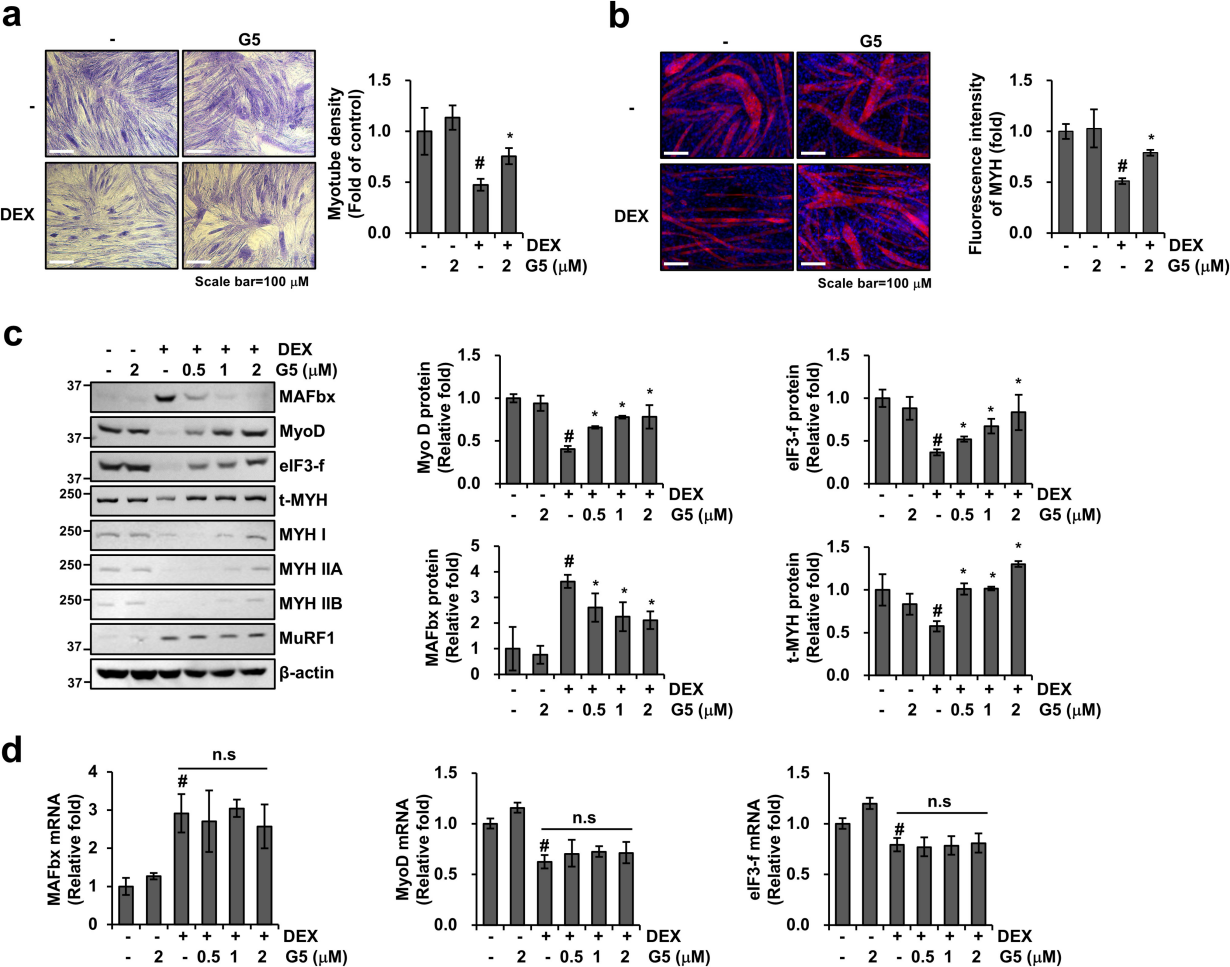
Effect of G5 on DEX‐induced muscle atrophy in C2C12 myotubes. (a–d) C2C12 myotubes were treated with G5, followed by DEX for 48 h. The cells were fixed and stained with Giemsa stain (a). IF was performed using an Alexa Fluor 546‐conjugated MYH antibody, and nuclei were stained with DAPI (b). Protein (c) and mRNA (d) levels were determined using western blotting and qPCR, respectively. ^#^ *p* < 0.01 compared to control. *p < 0.05 compared to DEX.

Ubiquitin isopeptidase inhibitor I (G5) is a pharmacological inhibitor that selectively targets YOD1 [26]. G5 rescued DEX‐induced reduction in myotube density and MYH fluorescence‐positive cells (Figure 5a,b). In addition, G5 prevented the MAFbx‐induced downregulation of MyoD and eIF3‐f expression by DEX, indicating the decrease of MYH isoform expression (Figure 5c). However, G5 did not affect the DEX‐induced increase in *MAFbx* mRNA levels and decrease in *MyoD* and *eIF3‐f* mRNA levels, indicating the post‐translation regulation of their expression by MAFbx (Figure 5d). Furthermore, the DEX‐induced decrease in protein synthesis was prevented by G5 through sustained phosphorylation of Akt, p70S6K and 4EBP1 (Figure S2b). These data demonstrate the possibility of using a YOD1 inhibitor as a therapeutic agent to improve muscle atrophy.

### 3.6 G5 Improves DEX‐Induced Muscle Atrophy In Vivo

**FIGURE 6:**
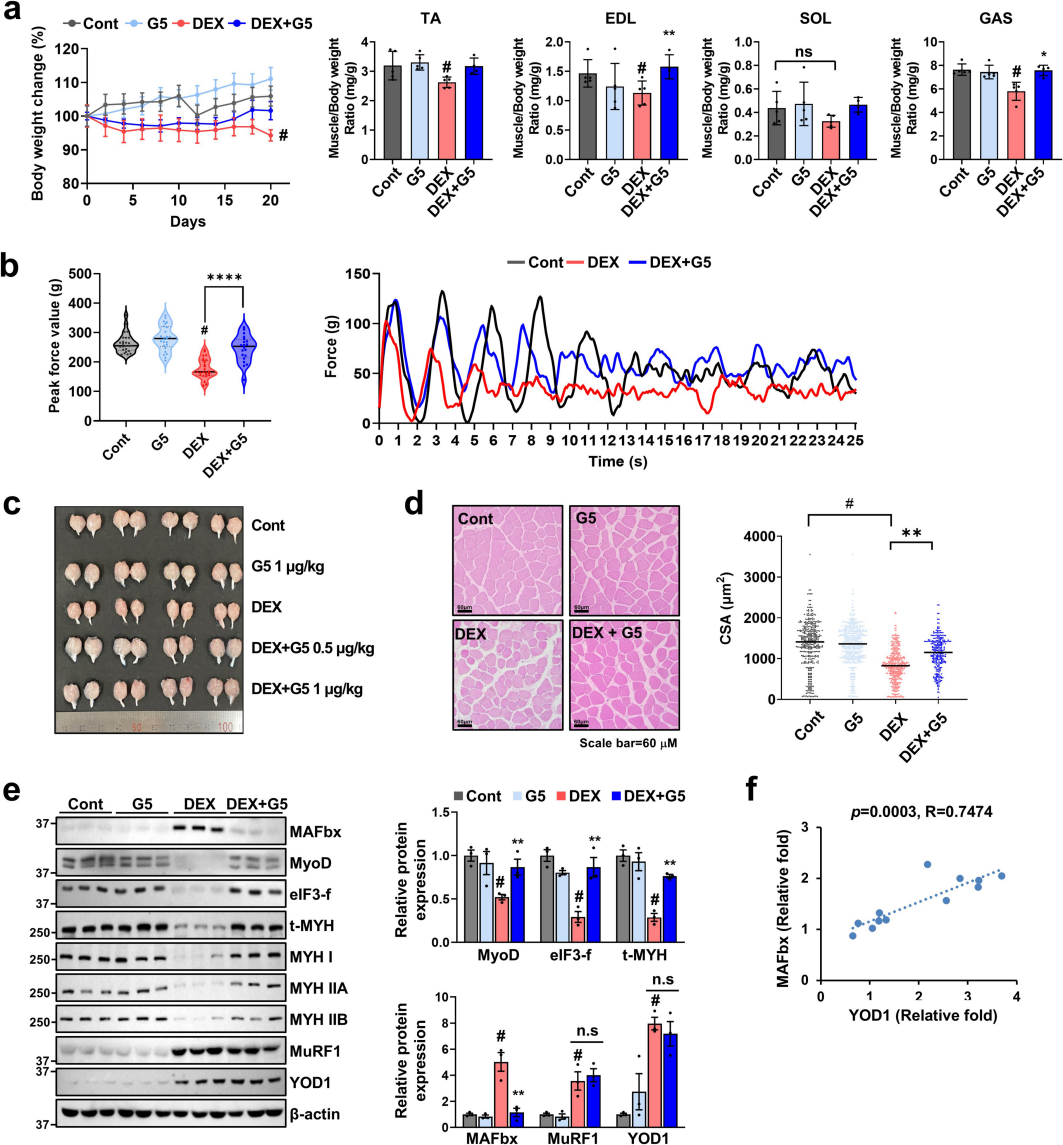
Effect of G5 on DEX‐induced muscle atrophy in mice. (a) The changes in body weight during the experimental periods. The muscle tissue weights were measured and normalized to body weight (*n* = 5). (b) Grip strength was represented by the peak force value and the total measurement duration (25 s). (c) Representative image of size isolated from GAS muscles. (d) Representative image of cross‐sectional area (CSA) of myofibers isolated from GAS muscles using haematoxylin and eosin (H&E) staining. (e) Protein levels was determined using western blotting. The band intensity of proteins was analysed using ImageJ. (f) Correlation analysis between YOD1 and MAFbx protein expression in muscle tissues of DEX‐injected mice (*n* = 12). ^#^ *p* < 0.01 compared to control. *p < 0.05 compared to DEX. **p < 0.01 compared to DEX. ****p < 0.001 compared to DEX.

Next, we established a mice model of DEX‐induced muscle atrophy to verify the protective effect of G5 against muscle wasting. The body and muscle (EDL, SOL and GAS) weights were found to be significantly reduced in the DEX‐injected group than in the control group; however, G5 recovered DEX‐induced loss of body and GAS muscle weight (Figure 6a). In the grip strength test, DEX showed reduced grip force. In contrast, G5 showed improved grip strength reduced by DEX, comparable with that of the control group (Figure 6b). Additionally, in the levels of serum creatine phosphokinase (CPK), a marker of cardiac and skeletal muscle damage, G5 inhibited DEX‐induced CPK levels (Figure S3a). Next, we assessed muscle fibre size in the GAS using H&E staining and observed that the CSA of the muscle fibres in the DEX group was markedly smaller than that in the control group. G5 attenuated the effects of DEX on the CSA of the GAS (Figure 6c,d). As observed in vitro, DEX increased MAFbx protein expression, which was reversed by G5 treatment, eventually resulting in the maintenance of MyoD and eIF3‐f protein levels in the muscle tissue of mice (Figure 6e). Furthermore, G5 reversed DEX‐decreased phosphorylation of Akt, p70S6K and 4EBP1 (Figure S3b). Consistently, YOD1 protein level was positively correlated with MAFbx protein level in GAS muscle tissues of DEX‐injected mice (Figures 6f and S3d). These data reveal that G5 effectively protected against DEX‐induced muscle atrophy.

### 3.7 G5 Ameliorates Sciatic Denervation (NTX)‐Induced Muscle Atrophy

**FIGURE 7:**
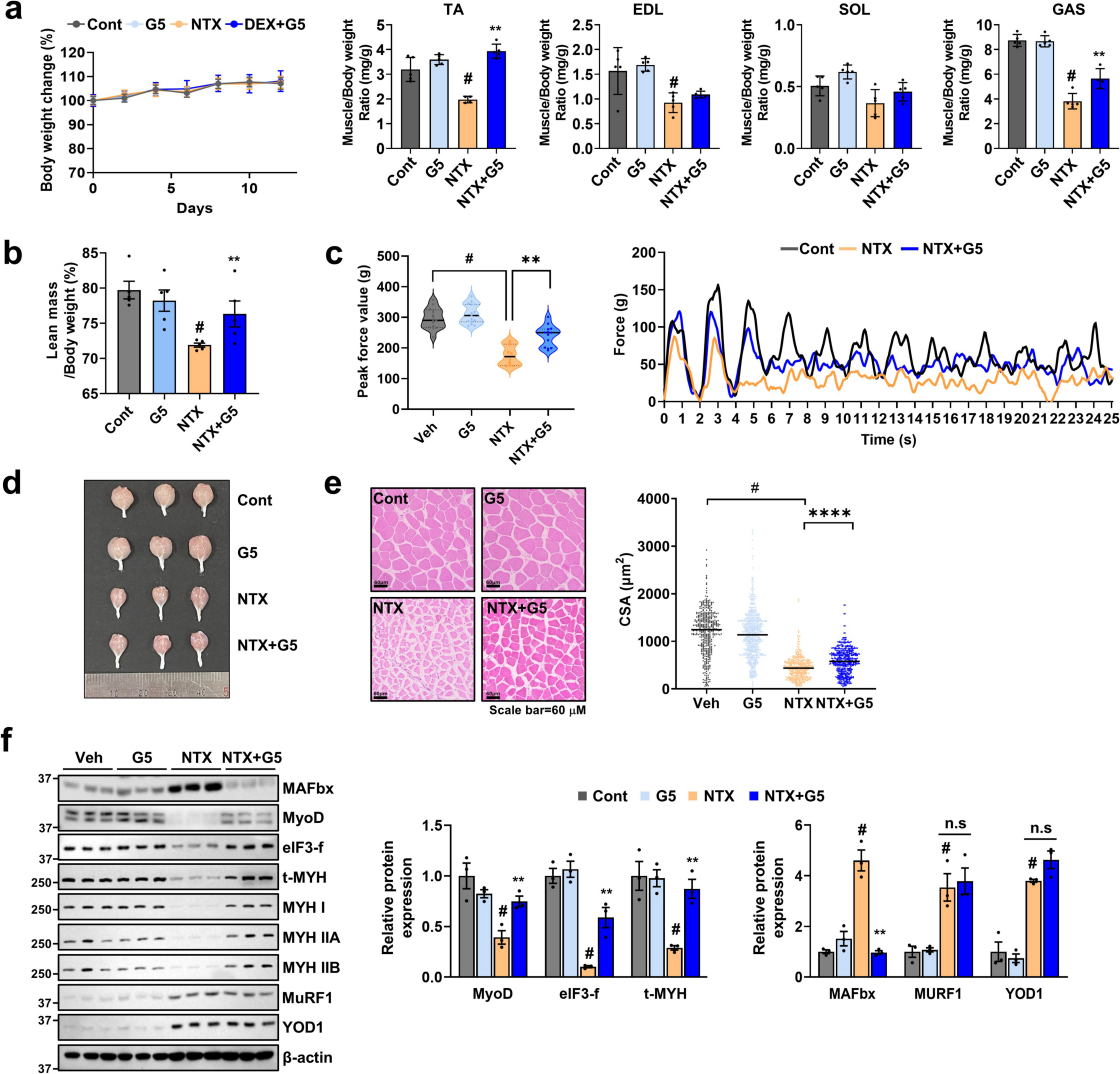
Effect of G5 on NTX‐induced muscle atrophy in mice. (a) The changes in body weight during the experimental periods. The muscle tissue weights were measured and normalized to body weight (*n* = 5) (b) Body composition showing lean body mass and whole‐body weight ratio (%). (c) Grip strength was represented by the peak force value and the total measurement duration (25 s). (d) Representative image of size isolated from GAS muscles. (e) Representative image of CSA of myofiber isolated from GAS muscles using H&E staining. (f) Protein levels was determined using western blotting. The band intensity of proteins was analysed using ImageJ. *p < 0.05 compared to DEX. **p < 0.01 compared to DEX. ****p < 0.001 compared to DEX.

In a mouse model of sciatic neurectomy (NTX)‐induced muscle atrophy, there were no significant differences in body weight between the four groups (Figure 7a). The weights of the TA, EDL, SOL and GAS muscles were significantly reduced in the NTX group, whereas those of the TA and GAS muscles were improved in the NTX + G5‐treated group (Figure 7a). Body composition analysis revealed that G5 reverses the loss of lean mass induced by NTX (Figure 7b). Both the peak force values and overall grip strength of the NTX group were lower than those of the control group; however, the G5 group alleviated NTX‐decreased grip strength (Figure 7c). Histological analysis showed a significant reduction in the CSA of muscle fibres in the NTX group, whereas G5 attenuated the effect of NTX (Figure 7d,e). In addition, G5 treatment suppressed the NTX‐induced increase of MAFbx expression and decrease of its substrate proteins (MyoD and eIF3‐f), as well as the reduced phosphorylation of Akt, p70S6K and 4EBP1 (Figures 7f and S3c). YOD1 protein expression is consistently upregulated across all muscles (TA, EDL and SOL) in response to both DEX treatment and NTX (Figure S3e). These results indicated that G5 ameliorates the loss of mass and muscle atrophy in the NTX‐induced mouse model.

### 3.8 YOD1 Expression Is Associated With Reduced Myofiber Size and Proteostasis Transcriptional Signatures in Human Skeletal Muscle

**FIGURE 8:**
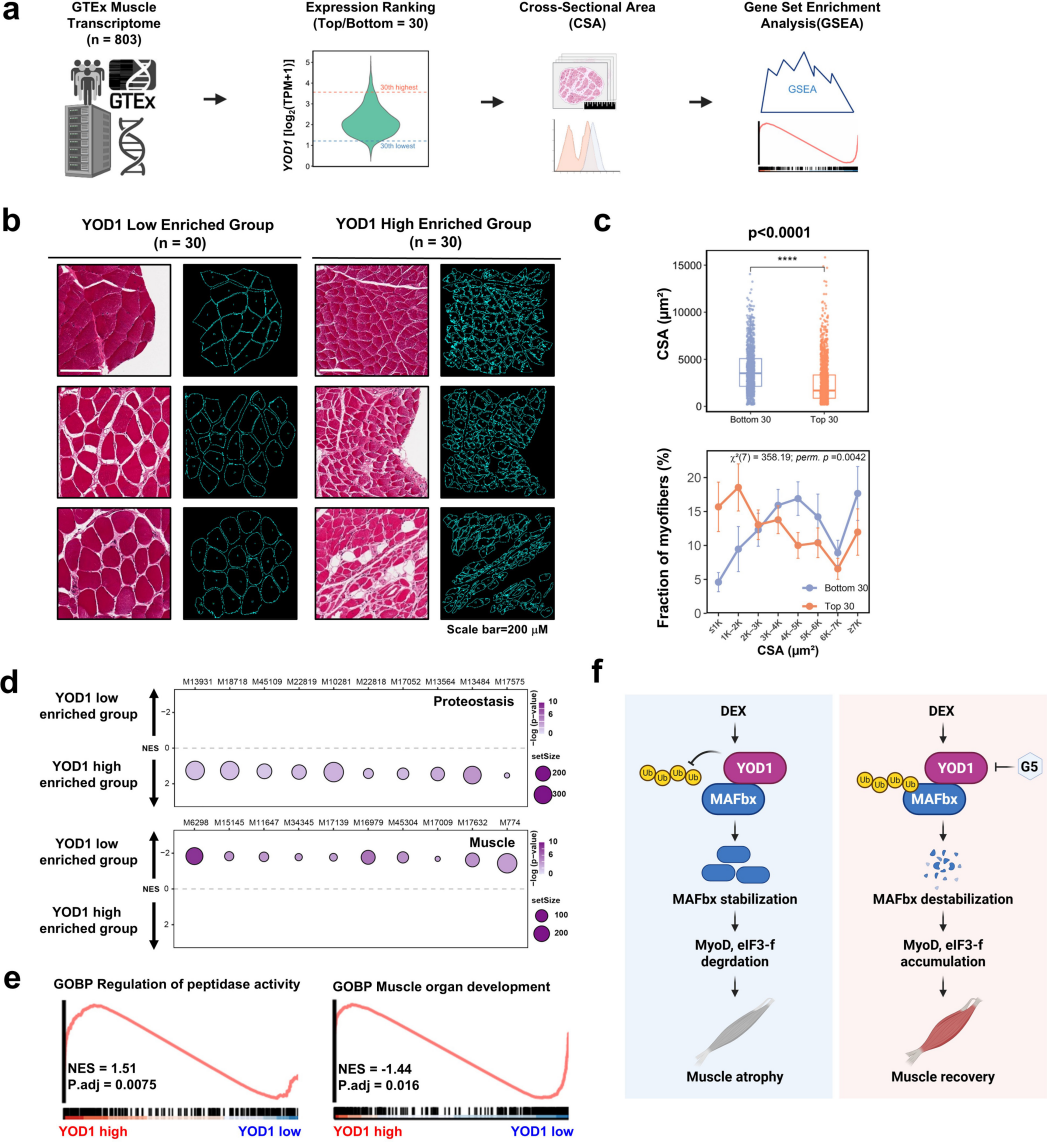
Human skeletal muscle with high YOD1 expression shows reduced fibre size and enrichment of subcellular stress–proteostasis pathways. (a) Schematic of the analysis workflow: GTEx skeletal muscle transcriptomes (*n* = 803) were ranked by YOD1 expression; the top 30 and bottom 30 donors were selected; CSA was quantified from matched H&E images, and gene set enrichment analysis (GSEA) was performed. (b) Representative H&E images and segmentation masks from low‐ and high‐YOD1 groups. Scale bars = 200 μm. (c) Box plots of CSA showing median CSA in the low‐ and high‐YOD1 groups. Each dot represents the CSA of a single myofiber quantified from H&E‐stained sections. The boxplot depicts the median (horizontal line), interquartile range (box) and overall distribution (whiskers) of myofiber CSA within each group. Distribution curves of CSA illustrating the frequency of myofibers across defined size bins in the low‐ and high‐YOD1 groups. Statistical comparison of the overall distributions between groups was performed using a chi‐square test. (d, e) GSEA of low‐ and high‐YOD1 groups. Bubble plots showing enriched gene sets. Bubble size indicates gene set size; colour intensity represents –log10p (d). Representative enrichment curves of GOBP identified in GSEA (e). (f) Schematic diagram of YOD1 inhibition‐mediated muscle recovery mechanisms.

To extend our discovery to the human context, we used the GTEx resource, in which transcriptomic and histological data are derived from the same skeletal muscle tissue to evaluate the relationship between YOD1 expression and myofiber CSA. From 803 skeletal muscle transcriptomes, we selected the 30 donors each with the highest and lowest YOD1 expression levels and quantified CSA from their matched H&E images (Figure 8a). Representative H&E‐stained sections with automated segmentation overlays revealed clear morphological differences between the groups (Figure 8b). The low‐YOD1 group exhibited larger polygonal fibres with relatively uniform size, whereas the high‐YOD1 group displayed smaller and more irregular fibres (Figure 8b). Quantitative analysis confirmed these observations, with box plots demonstrating a significantly lower median CSA in the high‐YOD1 group than in the low‐YOD1 group (Figure 8c). Distributional analysis further showed that high‐YOD1 donors were enriched for smaller fibres (< 2000 μm^2^) and exhibited a lower proportion of larger fibres (5000–7000 μm^2^), resulting in a CSA profile that was left‐shifted in high‐YOD1 donors relative to low‐YOD1 donors (*χ* ^2^(7) = 358.19, permutation *p* = 0.0042 Figure 8c).

To complement these histological findings and determine the molecular signatures underlying the differences associated with YOD1 expression, we performed an unbiased GSEA. This analysis comparing high‐ and low‐YOD1 donors revealed coherent enrichment of proteostasis‐related pathways, including the regulation of protein stability, ubiquitination and autophagy. In contrast, muscle structural and developmental programmes were relatively downregulated in the high‐YOD1 group (Figure 8d and Table S4). Representative enrichment curves illustrated opposing patterns: GOBP regulation of peptidase activity (NES = 1.51; adjusted *p* = 0.0075) was positively enriched, whereas GOBP muscle organ development was negatively enriched (NES = −1.44; adjusted *p* = 0.046; Figure 8e). Taken together, these results demonstrate that elevated YOD1 expression in human skeletal muscle is associated with reduced fibre size and transcriptional signatures of proteostasis, suggesting that the phenomena observed in vitro and in vivo may also be evident in humans.

## 4 Discussion

We found that genetic and pharmacological inhibition of YOD1 blocked DEX‐induced muscle atrophy both in vitro and in vivo. Mechanistic studies showed that YOD1 directly stabilizes the MAFbx E3 ligase by reducing its ubiquitination. The UBX domain of YOD1 interacts with the LZ domain of MAFbx, removing the ubiquitin chain at K48 on MAFbx. Additionally, the YOD1‐specific inhibitor G5 alleviated DEX‐ and denervation‐induced muscle atrophy in mice. These findings present YOD1 as a novel target for the treatment of muscle pathogenesis (Figure 8f).

Previous studies indicate that DUBs play diverse and context‐dependent roles in skeletal muscle homeostasis. UCHL1 has been reported to promote myoblast proliferation and inhibit myotube formation by regulating myogenin levels. Although UCHL1 expression accumulates in injured or denervated muscles, and muscle‐specific UCHL1 knockout only ameliorates injury‐induced skeletal muscle damage in mice, it fails to reverse denervation‐induced muscle atrophy [27, 28]. Moreover, OTUD7A is linked with neuromuscular disorders, and its loss leads to muscle hypotonia and intellectual disability in both mice and humans [29]. Nevertheless, the molecular mechanisms by which OTUD7A mediates muscle weakness remain poorly understood. Similarly, USP19 has been implicated in muscle wasting; however, its direct substrate has yet to be identified [22]. In contrast to these findings, our study establishes YOD1 as a mechanistically defined DUB that targets the muscle‐specific E3 ligase, MAFbx. Unlike UCHL1, which has limited impact on atrophy, or OTUD7A and USP19, whose targets remain elusive, we demonstrated that YOD1 interacts with and deubiquitinates MAFbx, thereby stabilizing MAFbx and promoting the degradation of its downstream targets, such as MyoD and eIF3‐f, ultimately accelerating the progression of muscle atrophy. Therefore, these findings clarify the unique role of YOD1 within the wider DUB landscape and provide a novel mechanistic insight into post‐translational regulation of muscle‐specific E3 ligases during muscle wasting.

YOD1 (also known as OTUD2) is a highly conserved deubiquitinating enzyme primarily involved in cancer development. YOD1 has a tumour‐regulatory function by deubiquitinating and stabilizing different proteins, such as p53, CDK1, β‐catenin and YAP/TAZ, associated with the progression of various cancers [30, 31, 32, 33]. Moreover, YOD1 dysregulation can affect various diseases including cardiovascular and neurodegenerative diseases [34]. However, the role of YOD1 in muscle homeostasis remains unclear. Our results indicated that YOD1 inhibition alleviates the DEX‐induced decrease in myotube density and MYH protein expression (Figures 2 and 5). Consistent with the in vitro findings, a specific inhibitor of YOD1 (G5) improved muscle function and restored muscle tissue that was reduced by atrophy in DEX‐induced and NTX‐induced mice models (Figures 6 and 7). Furthermore, we presented the novel finding that YOD1 directly binds to and deubiquitinates MAFbx, indicating sustained protein stability of MAFbx. YOD1 knockdown increased MAFbx ubiquitination, whereas YOD1 overexpression decreased its ubiquitination. However, this effect was not observed with the catalytic‐site mutant (C160S) (Figure 3). Moreover, YOD1 removed the polyubiquitin chain at K48 of MAFbx (Figure 4f). Although our findings suggest that YOD1 functions as a direct regulator of MAFbx, we cannot entirely exclude the possibility of an indirect regulatory mechanism, such as modulation of other E3 ligases responsible for MAFbx degradation. To investigate this possibility, we explored whether YOD1 affects several well‐established muscle‐related E3 ligases, including NEDD4, Trim32 and Fbxo30 (MUSA1). However, knockdown of YOD1 did not alter the protein levels of these E3 ligases under DEX treatment (data not shown). Although further research is required on the identification of E3 ligases for MAFbx and the mutual regulation of these E3 ligases and YOD1, our results to date indicate that MAFbx stabilization by YOD1 is caused through direct deubiquitination.

Research is ongoing on the target substrates of MAFbx and MuRF1 E3 ligases during muscle atrophy. Major substrates of MAFbx include MyoD, eIF3‐f and myogenin [35, 36]. In contrast, MuRF1 primarily targets structural proteins such as troponin I, myosin heavy and light chains and myosin‐binding protein C [37, 38, 39]. Recently, Baehr et al. employed di‐glycine ubiquitin remnant proteomics to expand the MuRF1 ubiquitylome and identified the ubiquitination of proteins including p62, VCP and several metabolic enzymes upon MuRF1 overexpression [40]. Despite extensive knowledge regarding the substrates of E3 ligases in muscle atrophy, the molecular mechanisms governing the post‐translational regulation of E3 ligases themselves have yet to be fully elucidated. To address this knowledge gap, we identified YOD1 as a novel post‐translational regulator that critically maintains MAFbx stability. In a DEX‐mediated muscle atrophy model, MAFbx destabilization by YOD1 inhibition was confirmed to increase not only the expression of MyoD and eIF3‐f proteins but also myosin heavy chain proteins (Figure 6e). YOD1 inhibition‐mediated t‐MYH increase is likely induced by a mechanism independent of MuRF1, as no changes in MuRF1 expression were observed. Further investigation into how YOD1 inhibition‐mediated t‐MYH increase occurs is required. Therefore, MyoD and eIF3‐f expression, target substrates of MAFbx, were altered according to YOD1‐dependent MAFbx regulation. For the first time, we identified YOD1 as a regulator of MAFbx and uncovered the mechanisms underlying its post‐translational regulation.

In conclusion, the present study provides the first evidence that the deubiquitinating enzyme YOD1 plays a critical role in maintaining MAFbx stability during post‐translational modifications. These results suggest that YOD1 can be an effective and selective genetic target for the treatment or improvement of muscle atrophy.

## Funding

This work was supported by the Bio&Medical Technology Development Program of the National Research Foundation (NRF) funded by the Korean government (MSIT) (RS‐2025‐18492970) and National Research Foundation of Korea (NRF) grant funded by the Korean Government (MSIP) (RS‐2024‐00453109).

## Ethics Statement

All animal experiments were approved by the Institutional Animal Care Committee of Keimyung University (approval number: KM‐2024‐11R1).

## Conflicts of Interest

The authors declare no conflicts of interest.

## Supporting information


**FIGURE S1:** Investigation of expression of deubiquitinating enzymes (DUBs) during muscle atrophy. (a) The heatmap generated from the analysis of GEO datasets GSE159952 and GSE149453 identified USP, JAMM, MJD, SENP and UCH family DUBs. (b) Protein expression levels of OTU family DUBs in GAS muscle tissue of mice treated with or without DEX for 3 weeks. (c) Protein expression levels of MYH, MAFbx and YOD1 in TA, EDL and SOL muscle tissue of mice treated with or without DEX for 3 weeks. (d) Protein expression levels of MYH, MAFbx, MURF1 and YOD1 on treatment of DEX, palmitic acid (PA), TNF‐α, H_2_O_2_ or serum‐free for 48 h in C2C12 myotube.
**FIGURE S2:** Inhibition of YOD1 regulates protein synthesis capacity during DEX‐induced muscle atrophy. (a) C2C12 myotubes were treated with G5, followed by DEX for 48 h. (b) C2C12 myotubes transfected with control or YOD1 siRNA were treated with DEX for 48 h. The incorporation of puromycin into newly synthesized proteins and protein expression was detected by western blotting.
**FIGURE S3:** Effect of G5 on AKT–mTOR signalling in DEX‐ and NTX‐induced muscle atrophy in mice. (a) CPK levels in serum (*n* = 5). (b,c) Protein expression of AKT signalling pathway (p‐AKT, AKT, p‐p70^S6K^, p70^S6K^, p‐4EBP1 and 4EBP1) and ACTN3 in GAS muscle tissue of DEX‐ (b) or NTX (c) ‐induced muscle atrophy mice. (c) Protein expression of MAFbx and YOD1 in GAS muscle tissue of DEX treatment. (e) Protein expression levels of YOD1 and t‐MYH in TA, EDL and SOL muscle tissue of mice by DEX and NTX. ^#^ *p* < 0.01 compared to control.
**Table S1:** The information of siRNAs and plasmids.
**Table S2:** The information of antibodies and chemicals.
**Table S3:** The sequence of qPCR primers.
**Table S4:** Gene sets enriched in high‐ vs. low‐*YOD1* donors identified by GSEA. Significantly enriched pathways are grouped into two major categories: proteostasis and muscle. Each entry includes the MSigDB gene set ID and the corresponding GO annotation term.

## Data Availability

Data sharing not applicable to this article as no datasets were generated or analysed during the current study.
