# Supplementary material for: Deubiquitinase YOD1 Inhibition Suppresses DEX‐ and Denervation‐Induced Muscle Atrophy Through MAFbx Destabilization
Source: J Cachexia Sarcopenia Muscle. 2026 Apr 29;17(3):e70300. doi: 10.1002/jcsm.70300 (PMC13126240; doi:10.1002/jcsm.70300)
Supplement: Supplementary file 1 — FIGURE S1: Investigation of expression of deubiquitinating enzymes (DUBs) during muscle atrophy. (a) The heatmap generated from the analysis of GEO datasets GSE159952 and GSE149453 identified USP, JAMM, MJD, SENP and UCH family DUBs. (b) Protein expression levels of OTU family DUBs in GAS muscle tissue of mice treated with or without DEX for 3 weeks. (c) Protein expression levels of MYH, MAFbx and YOD1 in TA, EDL and SOL muscle tissue of mice treated with or without DEX for 3 weeks. (d) Protein expression levels of MYH, MAFbx, MURF1 and YOD1 on treatment of DEX, palmitic acid (PA), TNF‐α, H2O2 or serum‐free for 48 h in C2C12 myotube. FIGURE S2: Inhibition of YOD1 regulates protein synthesis capacity during DEX‐induced muscle atrophy. (a) C2C12 myotubes were treated with G5, followed by DEX for 48 h. (b) C2C12 myotubes transfected with control or YOD1 siRNA were treated with DEX for 48 h. The incorporation of puromycin into newly synthesized proteins and protein expression was detected by western blotting. FIGURE S3: Effect of G5 on AKT–mTOR signalling in DEX‐ and NTX‐induced muscle atrophy in mice. (a) CPK levels in serum (n = 5). (b,c) Protein expression of AKT signalling pathway (p‐AKT, AKT, p‐p70S6K, p70S6K, p‐4EBP1 and 4EBP1) and ACTN3 in GAS muscle tissue of DEX‐ (b) or NTX (c) ‐induced muscle atrophy mice. (c) Protein expression of MAFbx and YOD1 in GAS muscle tissue of DEX treatment. (e) Protein expression levels of YOD1 and t‐MYH in TA, EDL and SOL muscle tissue of mice by DEX and NTX. # p < 0.01 compared to control. Table S1: The information of siRNAs and plasmids. Table S2: The information of antibodies and chemicals. Table S3: The sequence of qPCR primers. Table S4: Gene sets enriched in high‐ vs. low‐YOD1 donors identified by GSEA. Significantly enriched pathways are grouped into two major categories: proteostasis and muscle. Each entry includes the MSigDB gene set ID and the corresponding GO annotation term. [file JCSM-17-e70300-s001.docx]

**Supplementary Information**

**Figure S1-3**

**Tables S1-4**

**Reference S1-5**

**Measurement of Protein Synthesis**To evaluate global protein synthesis in vitro, we conducted Surface Sensing of Translation (SUnSET) assay. The cells were treated with puromycin (50 μg/mL) for 30 minutes at 37°C. After incubation, cells were washed with PBS and lysed using RIPA buffer supplemented with protease inhibitors. Total protein concentration was determined by BCA assay, and equal amounts of protein were resolved by SDS-PAGE. Incorporated puromycin was detected by immunoblotting using an anti-puromycin monoclonal antibody.

**
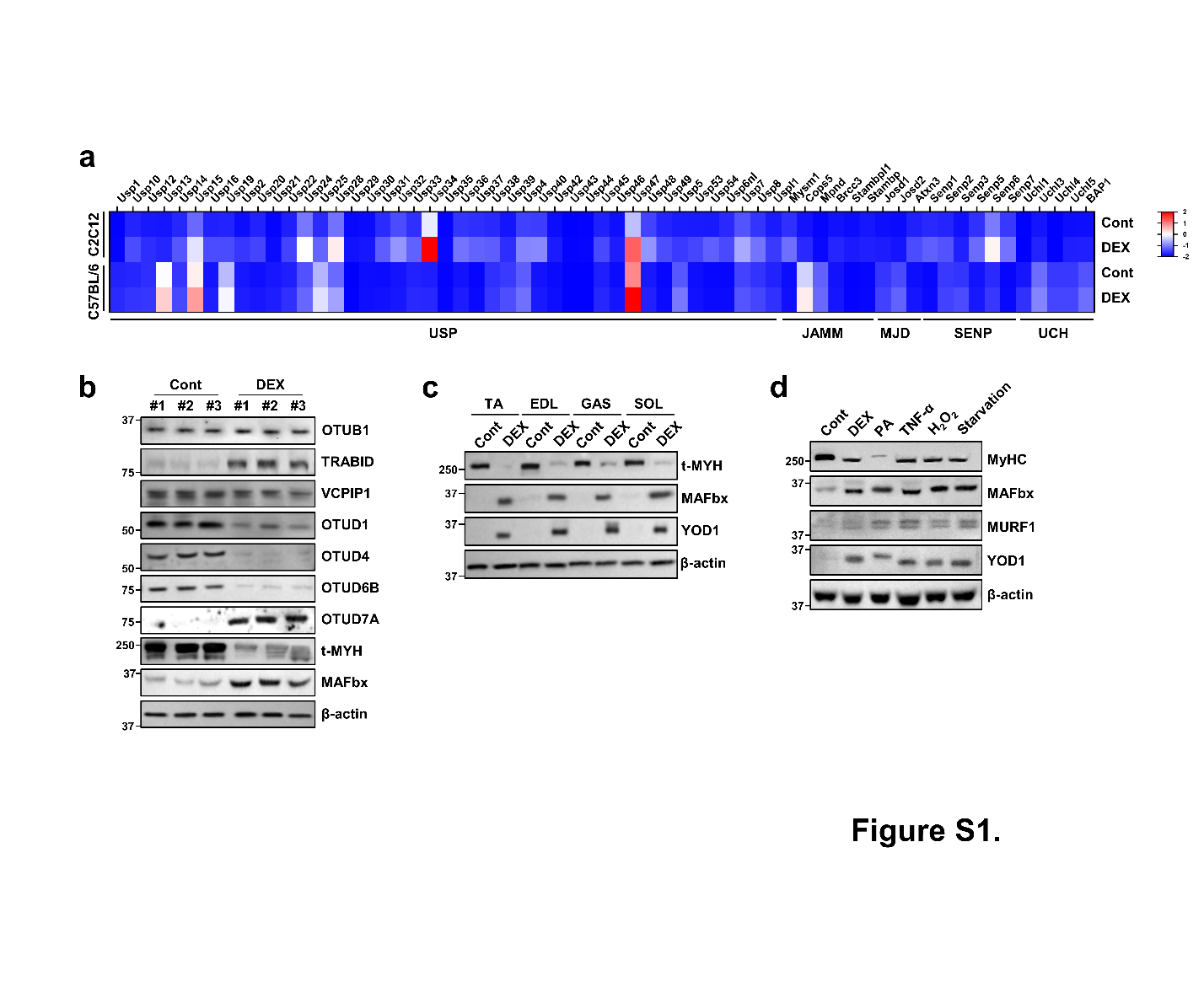
**

**FIGURE S1** Investigation of expression of deubiquitinating enzymes (DUBs) during muscle atrophy. (a) The heatmap generated from the analysis of GEO datasets GSE159952 and GSE149453 identified USP, JAMM, MJD, SENP and UCH family DUBs. (b) Protein expression levels of OTU family DUBs in GAS muscle tissue of mice treated with or without DEX for 3 weeks. (c) Protein expression levels of MYH, MAFbx, and YOD1 in TA, EDL, SOL muscle tissue of mice treated with or without DEX for 3 weeks. (d) Protein expression levels of MYH, MAFbx, MURF1, and YOD1 on treatment of DEX, palmitic acid (PA), TNF-α, H_2_O_2_, or serum-free for 48 h in C2C12 myotube.

**
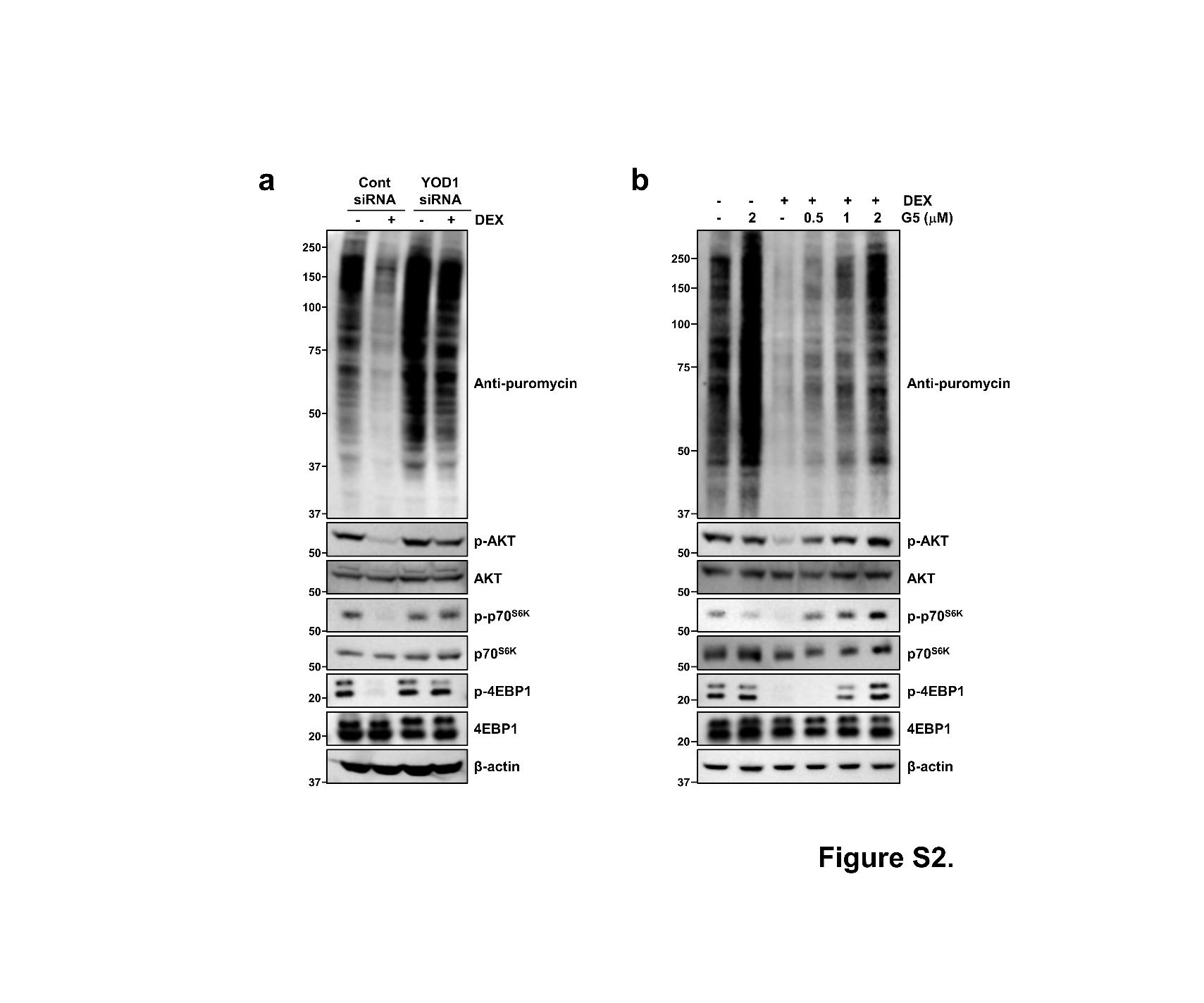
**

**FIGURE S2** Inhibition of YOD1 regulates protein synthesis capacity during DEX-induced muscle atrophy. (a) C2C12 myotubes were treated with G5, followed by DEX for 48 h. (b) C2C12 myotubes transfected with control or YOD1 siRNA were treated with DEX for 48 h. The incorporation of puromycin into newly synthesized proteins and protein expression were detected by western blotting.


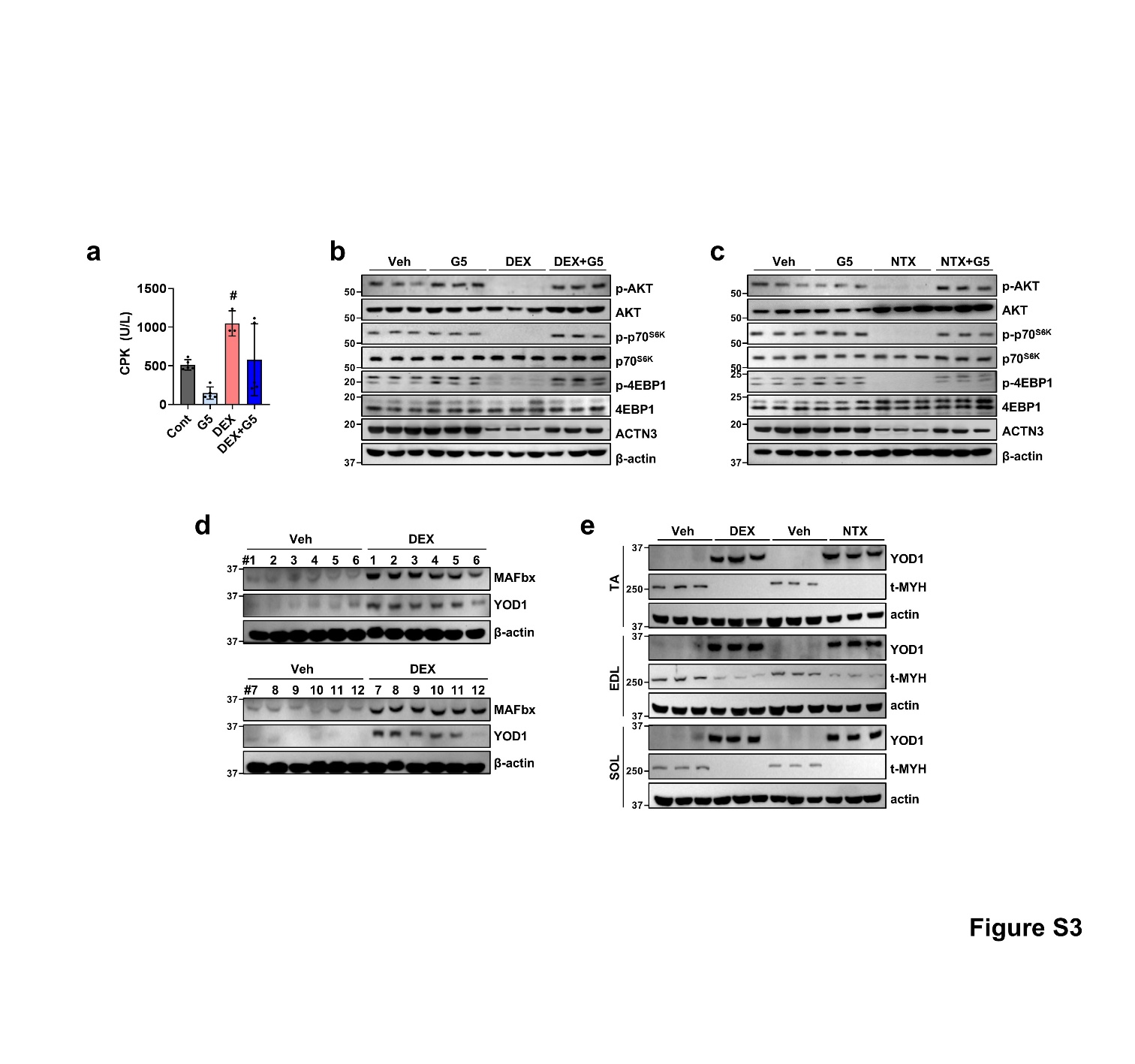


**FIGURE S3** Effect of G5 on AKT-mTOR signaling in DEX- and NTX-induced muscle atrophy in mice. (a) CPK levels in serum (n=5). (b,c) Protein expression of AKT signaling pathway (p-AKT, AKT, p-p70^S6K^, p70^S6K^, p-4EBP1, and 4EBP1) and ACTN3 in GAS muscle tissue of DEX- (b) or NTX (c) -induced muscle atrophy mice. (c) Protein expression of MAFbx and YOD1 in GAS muscle tissue of DEX treatment. (e) Protein expression levels of YOD1 and t-MYH in TA, EDL, and SOL muscle tissue of mice by DEX and NTX. ^#^*p* < 0.01 compared to control.

**Table S1.** The information of siRNAs and plasmids.

| Name | Sequence (5’-3’) | |
| --- | --- | --- |
| GFP (control) siRNA | GUUCAGCGUGUCCGGCGAG | |
| DUBs | | |
| OTUB1 siRNA | GACAACAUCUAUCAACAGA | |
| OTUB2 siRNA | CCGUUUACCUGCUCUAUAA | |
| TRABID siRNA | GAAGAAGAAUCUCCAAUUA | |
| OTUD1 siRNA | ACGAGAAGCUGGCCCUAUA | |
| OTUD4 siRNA | GGGUAGGACAAGUGGAAAU | |
| OTUD5 siRNA | GAAAGCAUUGCAUGGACUA | |
| OTUD6B siRNA | GAAACUUGCUCAAAUAUUG | |
| OTUD7 siRNA | UGUCCUAGCCCAUAUAUUA | |
| VCPiP1 siRNA | GAGAAGCUCUGGUGAUUAU | |
| YOD1 siRNA | GGGAGGAGCAAUAGAGAUA | |
| Constructs | Supplier | Cat No. |
| pcDNA3.1 (+) | Thermo Fisher Scientific | Cat# V79020 |
| HA-Ubiquitin | Addgene | Cat# 18712 |
| eGFP-YOD1 | Addgene | Cat# 85664 |
| eGFP-YOD1 C160S | Addgene | Cat# 85666 |
| eGFP-YOD1 ΔZn | Cloning | N/A |
| eGFP-YOD1 ΔUBX | Cloning | N/A |
| FLAG-MAFbx | Cloning | N/A |
| FLAG-MAFbx ΔF-box | Cloning | N/A |
| FLAG-MAFbx ΔNLS2 | Cloning | N/A |
| FLAG-MAFbx ΔC-term | Cloning | N/A |
| FLAG-MAFbx K29R | Cloning | N/A |
| FLAG-MAFbx K48R | Cloning | N/A |
| FLAG-MAFbx K267R | Cloning | N/A |

**Table S2.** The information of antibodies and chemicals

| Antibodies | Company | | Cat # |
| --- | --- | --- | --- |
| β-actin | Sigma-Aldrich | | Cat# A2228 |
| FLAG | Sigma-Aldrich | | F1804 |
| t-MYH | Developmental Studies Hybridoma Bank | | MF20 |
| MYH I | Developmental Studies Hybridoma Bank | | BA-F8 |
| MYH IIA | Developmental Studies Hybridoma Bank | | BA-D5 |
| MYH IIB | Developmental Studies Hybridoma Bank | | BF-F3 |
| OTUD1 | Proteintech | | 29921-1-AP |
| YOD1 | Proteintech | | 25370-1-AP |
| OTUB1 | Santa Cruz Biotechnology | | sc-130458 |
| TRABID | Santa Cruz Biotechnology | | sc-374377 |
| VCPIP1 | Santa Cruz Biotechnology | | sc-515291 |
| p-AKT | Cell Signaling Technology | | #9271 |
| AKT | Cell Signaling Technology | | #9272 |
| p-p70S6K | Cell Signaling Technology | | #9205 |
| p70S6K | Cell Signaling Technology | | #9202 |
| p-4EBP1 (Thr37/46) | Cell Signaling Technology | | #2855 |
| 4EBP1 | Cell Signaling Technology | | #9644 |
| GFP | Abcam | | ab290 |
| OTUD4 | Abcam | | ab106368 |
| MAFbx | Abcam | | ab168372 |
| OTUD7A | Abcam | | ab173988 |
| Anti-puromycin | Abcam | | ab315887 |
| eIF3-f | Santa Cruz Biotechnology | | sc-390413 |
| MURF1 | Santa Cruz Biotechnology | | sc-398608 |
| ACTN3 | Santa Cruz Biotechnology | | sc-17829 |
| Ub | Santa Cruz Biotechnology | | sc-8017 |
| HRP-Ub | Enzo Life Sciences | | BML-PW-0150-0100 |
| OTUD6B | NOVUS biologicals | | NBP1-85652 |
| Chemicals | | Source | Cat # |
| Ubiquitin isopeptidase inhibitor G5 | | MedChemExpress | HY-100738 |
| Dexamethasone | | Sigma-Aldrich | Cat# D4902 |
| CHX | | Sigma-Aldrich | Cat# 01810 |
| MG132 | | Sigma-Aldrich | Cat# M8699 |

**Table S3.** The sequence of qPCR primers.

| Name | Sequence (5’-3’) |
| --- | --- |
| MAFbx F | AAGGCTGTTGGAGCTGATAGCA |
| MAFbx R | CACCCACATGTTAATGTTGCCC |
| MYH I F | CTCAAGCTGCTCAGCAATCTATTT |
| MYH I R | GGAGCGCAAGTTTGTCATAAGT |
| YOD1 F | GTCAGCGAATCCTCGTTGGCTA |
| YOD1 R | CGCAGGTGAAGCTTTTGGTCTG |
| actin F | AGGCCCAGAGCAAGAGAGGTAC |
| actin R | AGGCATACAGGGACAGCACAGC |

**Table S4.** Gene sets enriched in high- vs. low-*YOD1* donors identified by GSEA. Significantly enriched pathways are grouped into two major categories: Proteostasis and Muscle. Each entry includes the MSigDB gene set ID and the corresponding GO annotation term.

|  | GSEA Systematic name | GO Term Name | Normalized enrichment score | P value |
| --- | --- | --- | --- | --- |
| Proteostasis | M13931 | GOMF Ubiquitin like protein ligase binding | 1.254162 | 1.5577E-2 |
|  | M18718 | GOBP Regulation of protein ubiquitination | 1.25936 | 1.7199E-2 |
|  | M45109 | GOBP Regulation of macroautophagy | 1.299277 | 4.0813E-2 |
|  | M22819 | GOBP Regulation of autophagy | 1.342949 | 1.0421E-2 |
|  | M10281 | GOBP Positive regulation of macroautophagy | 1.344608 | 3.707E-3 |
|  | M22818 | GOCC Autophagosome | 1.424787 | 7.383E-3 |
|  | M17052 | GOBP Positive regulation of peptidase activity | 1.43757 | 8.127E-3 |
|  | M13564 | GOBP Regulation of peptidase activity | 1.452128 | 1.775E-3 |
|  | M13484 | GOCC Autophagosome membrane | 1.513098 | 1.36E-4 |
| Muscle Function | M6298 | GOCC Contractile muscle fiber | -1.83721 | 1.71E-09 |
|  | M15145 | GOMF Structural constituent of muscle | -1.83082 | 9.46E-05 |
|  | M11647 | GOBP Skeletal muscle contraction | -1.7954 | 1.53E-4 |
|  | M34345 | GOCC Striated muscle thin filament | -1.77828 | 2.47E-4 |
|  | M17139 | GOCC Myofilament | -1.7714 | 3.59E-4 |
|  | M16979 | GOCC I band | -1.77029 | 2.71E-06 |
|  | M45304 | GOCC Sarcoplasmic reticulum | -1.76554 | 7.8E-05 |
|  | M17009 | GOCC Muscle myosin complex | -1.68321 | 5.43E-4 |
|  | M17632 | GOCC Sarcoplasmic reticulum | -1.62315 | 1.11E-4 |
|  | M774 | GOBP Muscle organ development | -1.43568 | 4.19E-4 |

**Supplementary References**

S1. W. Liang, F. Xu, L. Li, et al., "Epigenetic control of skeletal muscle atrophy," *Cell Mol Biol Lett* 29 (2024): 99.

S2. S. U. Seo, S. M. Woo, M. W. Kim, E. W. Lee, K. J. Min and T. K. Kwon, "Phosphorylation of OTUB1 at Tyr 26 stabilizes the mTORC1 component, Raptor," *Cell Death Differ* 30 (2023): 82–93.

S3. Y. Jo, M. K. Yeo, T. Dao, J. Kwon, H. S. Yi and D. Ryu, "Machine learning-featured Secretogranin V is a circulating diagnostic biomarker for pancreatic adenocarcinomas associated with adipopenia," *Front Oncol* 12 (2022): 942774.

S4. H. K. So, H. Kim, J. Lee, et al., "Protein Arginine Methyltransferase 1 Ablation in Motor Neurons Causes Mitochondrial Dysfunction Leading to Age-related Motor Neuron Degeneration with Muscle Loss," *Research (Wash D C)* 6 (2023): 0158.

S5. S. C. Bodine and J. D. Furlow, "Glucocorticoids and Skeletal Muscle," *Adv Exp Med Biol* 872 (2015): 145–176.
